# Severe dengue infection unmasking drug‐induced liver injury: Successful management with N‐acetylcysteine

**DOI:** 10.1002/ccr3.8578

**Published:** 2024-03-10

**Authors:** Naveen Gautam, Nishan Shrestha, Sanjeev Bhandari, Sabin Thapaliya

**Affiliations:** ^1^ Maharajgunj Medical Campus Kathmandu Nepal; ^2^ Department of Internal Medicine Tribhuvan University Teaching Hospital Kathmandu Nepal

**Keywords:** anti‐tubercular therapy, dengue, drug‐induced liver injury, N‐acetylcysteine

## Abstract

**Key Clinical Message:**

Clinicians in tuberculosis and dengue endemic regions should have heightened vigilance for drug‐induced liver injury (DILI) overlapping with active infections, enabling prompt recognition and life‐saving conservative management.

**Abstract:**

Severe dengue and drug‐induced liver injury (DILI) are significant independent risk factors for acute liver failure. The co‐occurrence of these conditions significantly complicates clinical management. Here, we describe the case of a 21‐year‐old Nepali female who developed acute liver failure during antitubercular therapy (ATT). The patient, presenting with fever and nausea after 3 weeks of ATT, subsequently received a diagnosis of severe dengue. Laboratory evidence indicated markedly elevated transaminases (AST 4335 U/L, ALT 1958 U/L), total bilirubin (72 μmol/L), and INR (>5). Prompt discontinuation of first‐line ATT, initiation of a modified ATT regimen, and N‐acetylcysteine (NAC) infusion facilitated the patient's recovery after a week of intensive care. This case underscores the potential for synergistic hepatotoxicity in regions where multiple endemic illnesses coincide. Early recognition of DILI, cessation of offending agents, and comprehensive intensive care are crucial interventions. While the definitive efficacy of NAC remains under investigation, its timely administration in these complex cases warrants exploration for its potential lifesaving benefits.

## 1 INTRODUCTION

Dengue fever poses a critical global health challenge, with annual infections estimated at 100–400 million across over 100 endemic countries.^1^, ^2^ Nepal has witnessed a dramatic surge in dengue cases, likely driven by accelerating urbanization, climate change, and international transmission.^3^ This trend creates significant strain on healthcare systems, as evidenced by Nepal's 9411 dengue cases and 8 deaths in 2023, following a peak of 54,784 cases and 88 deaths in 2022.^4^ Concurrently, drug‐induced liver injury (DILI) remains a serious complication of antitubercular therapy (ATT), with reported incidences of 2%–28%.^5^ Severe dengue, independently capable of precipitating acute liver failure through direct hepatotoxicity, likely presents a significant compounding risk in patients receiving ATT.^6^

Case reports^7^, ^8^ suggest complex interactions between dengue and hepatotoxic medications, potentially involving reduced clearance, aberrant hapten formation, and synergistic immunotargeting.^9^ While isolated dengue‐induced liver injury often resolves spontaneously, concomitant drug‐induced acute liver failure carries a grave prognosis.^10^ Beyond supportive care, N‐acetyl cysteine (NAC) holds promise in managing acute liver failure. In cases where liver function parameters (enzymatic, synthetic, and coagulation) fail to improve within 72 h despite intensive support, prompt transplant evaluation may be critical where resources permit.^11^

Here, we present a case of severe dengue infection exacerbating ATT‐induced liver injury. This report offers valuable insights into timely recognition and management of overlapping hepatic insults due to co‐occurring infections and medication‐related toxicities.

## 2 CASE HISTORY/EXAMINATION

A 21‐year‐old Nepali female presented to the emergency department (ED) of Tribhuvan University Teaching Hospital (TUTH), a tertiary care center in Nepal, with a 5‐day history of fever, myalgia, vomiting, and generalized edema. Her recent medical history included smear‐positive pulmonary tuberculosis, diagnosed via sputum acid‐fast bacilli testing 20 days prior. A four‐drug anti‐tuberculosis therapy (ATT) regimen of rifampicin (R), isoniazid (H), pyrazinamide (Z), and ethambutol (E) had resulted in initial symptomatic improvement.

In the context of the ongoing dengue outbreak, she was ordered dengue NS1 antigen testing and liver function tests (LFTs) 2 days prior to her TUTH presentation. These revealed dengue NS1 positivity and acute liver injury with marked transaminase elevation, prompting a working diagnosis of severe dengue complicated by anti‐tuberculosis drug‐induced liver injury (AT‐DILI). The patient was referred to our center for intensive care unit (ICU) management.

On initial ED evaluation, the patient appeared ill but conscious. Vital signs included hypotension (60/40 mmHg), tachycardia (94 beats/min), oxygen saturation 96% on room air, and afebrile state. Physical examination revealed scleral icterus, but no pallor, cyanosis, clubbing, lymphadenopathy, dehydration, or chronic liver disease stigmata. Abdomen was soft, non‐tender, without hepatosplenomegaly. Auscultation revealed bilaterally clear lungs.

## 3 METHODS

Prior to presentation at TUTH ED, the patient received intravenous (IV) fluids at a primary hospital, and her ATT regimen was discontinued. Due to suspected DILI, acetaminophen was withheld. A dramatic rise in alanine transaminase (ALT) from over 4000 U/L to greater than 14,000 U/L prompted the initiation of N‐acetylcysteine (NAC) infusion (150 mg/kg over 1 h, followed by 12.5 mg/kg/h for 4 h, then 6.25 mg/kg/h for 16 h) and referral to TUTH for ICU management.

Upon arrival at TUTH, laboratory evaluation revealed leukocytosis (TLC 16000/microliters), anemia (Hb 8.8 g/dL, hematocrit 28.1%), but a normal platelet count (175,000/cmm). Markedly elevated liver function tests (LFTs) were evident, including aspartate transaminase (AST) 4335 U/L, ALT 1958 U/L, direct bilirubin 20 μmol/L, and International Normalized Ratio (INR) exceeding 5. Concurrent dengue virus infection was confirmed serologically via positive IgM antibodies and NS1 antigen. Abdominopelvic ultrasonography (USG) demonstrated gallbladder thickening, left nephrolithiasis, and ascites. Testing for a broader panel of tropical diseases (RK39, toxoplasma, typhoid, scrub typhus, and brucellosis) was negative.

The patient was admitted to the ICU with a principal diagnosis of AT‐DILI exacerbated by severe dengue. Critical illness severity scores were significantly elevated: Sequential Organ Failure Assessment (SOFA) score 2, Acute Physiology And Chronic Health Evaluation (APACHE) score II 7, Model for End‐Stage Liver Disease (MELD) score 25, Child‐Pugh C and King's Criteria 1 positive. Transthoracic echocardiography confirmed preserved left ventricular function (ejection fraction 55%). Management of shock included noradrenaline infusion (titrated up to 0.05 mcg/kg/min) and aggressive volume expansion with 5 L of intravenous crystalloid boluses within the first 8 h. Oral digoxin (0.125 mg daily) was initiated for inotropic support.

Table 1 provides a detailed timeline of laboratory investigations during the ED assessment, 7‐day ICU admission, subsequent ward stay, and 2‐week follow‐up. Vitamin K (10 mg/d) and ursodeoxycholic acid (UDCA, 300 mg/d) were also administered. The ATT regimen was modified to second‐line agents (levofloxacin, amikacin, and ethambutol). Supraventricular tachycardia (SVT) developed within the first 24 h of ICU admission and was managed with intravenous digoxin (0.25 mg loading, 0.125 mg maintenance) and oral metoprolol (50 mg twice daily). The patient achieved hemodynamic stability and improved liver function, facilitating transfer to the ward on Day 8 and discharge on Day 9.

**TABLE 1:** Results of laboratory tests of the patient.

Name of test	Day of illness									Follow‐up visit day 14
	At ED	ICU admission							Shifted to ward	
		Day 1	Day 2	Day 3	Day 4	Day 5	Day 6	Day 7		
Hb (g/dL)	8.8	10.6	8.8	9.1	8.1	8.4	8.2	8.6	9	
Hct (%)	28.1	33.8	25.8	26.6	24.2	25.8	25.6	25.3	29.9	
WBC (×10^9^/L)	7900	8300	5700	28,600	19,900	7500	6700	7900	8800	
Platelet (×10^9^/L)	175,000	181,000	151,000	97,000	89,000	79,000	57,000	28,000	85,000 (manual)	156,000
AST (U/L)	4335	4430	2707	1885	871	394	225	153	110	59
ALT (U/L)	1958	2190	1701	1456	1129	821	655	533	453	68
ALP (U/L)	191	196	151	127	126	–	83	89	112	
Albumin (g/L)	19	22	16	19	17	–	19	21	29	
Total bilirubin (μmol/L)	72	87	88	120	107	94	–	–	99	
Direct bilirubin (μmol/L)	20	56	58	76	68	59	‐	‐	82	
Creatinine (μmol/L)	41	42	33	72	39	43	34	‐	43	
PT/INR (s)	>60/>5	>60/>5	48/3.8	45/3.75	20/1.66	17/1.41	15/1.25	14/1.16	‐	
NS1 antigen	Positive									
Dengue IgM	Positive									
Dengue IgG	Positive									

## 4 CONCLUSION AND RESULTS

Outpatient follow‐up at 2 weeks demonstrated significantly decreased transaminases (ALT 68 U/L, AST 59 U/L) and normalized blood counts. Further HRZE re‐initiation plans were evaluated pending continued LFT normalization.

## 5 DISCUSSION

This case underscores the complexity of managing severe dengue in the context of potential anti‐tuberculosis treatment‐induced liver injury (AT‐DILI), an increasingly relevant concern in endemic regions. Our patient met established DILI diagnostic criteria, including significantly elevated ALT and bilirubin (>2× ULN), in the absence of pre‐existing cirrhosis or alternate causes of organ failure.^12^

While her constellation of fever, myalgia, thrombocytopenia, and edema aligned with a severe dengue presentation during an active outbreak, the marked hepatic enzyme derangements and coagulopathy strongly suggested an acute superimposed liver insult. This was likely precipitated by the recent initiation of rifampin and isoniazid therapy, which is known to carry a risk of drug‐induced hepatitis in up to 20% of cases.^13^, ^14^ Pyrazinamide, while part of the regimen, has a lower hepatotoxicity profile (3.77%).^15^ Further research is crucial to delineate potential synergistic mechanisms at play between concurrent endemic infections and AT‐DILI. Clinicians treating tuberculosis must maintain a high index of suspicion for superimposed liver injury, particularly during the initial intensive treatment phase where strict adherence monitoring is vital.

Although severe dengue alone rarely causes fulminant hepatitis, increased hepatocellular permeability (induced by NS1‐mediated endothelial glycocalyx damage) likely predisposes these patients to heightened drug toxicity risks.^16^ First‐line ATT agents like rifampin and isoniazid rely on extensive hepatic metabolism and exhibit dose‐dependent hepatotoxicity profiles.^17^ We theorize that infection‐related hepatic dysfunction may have reduced clearance of these agents' metabolites, leading to toxic accumulation.^18^ Additionally, the aberrant T‐cell activation characteristic of severe dengue could facilitate immune‐mediated hepatotoxicity via cross‐reactivity.^19^

Given the critical condition and high mortality risk (up to 13%) associated with severe dengue,^20^ our initial focus was on shock management and addressing coagulopathy. Prompt discontinuation of the suspected offending ATT agents (rifampin and isoniazid) was coupled with the initiation of N‐acetylcysteine (NAC). Despite limited definitive evidence outside of acetaminophen overdose,^21^ NAC is postulated to provide cytoprotective benefits in acute liver failure via replenishing glutathione, scavenging free radicals, and bolstering hepatic regeneration.^22^ Studies indicate improved survival with NAC in severe dengue with liver failure.^22^, ^23^ While evidence remains scarce, ursodeoxycholic acid's (UDCA) role in idiosyncratic DILI deserves exploration based on existing systematic reviews showing favorable biochemical parameters.^24^ Further high‐quality randomized controlled trials are necessary to determine whether regimens combining NAC and UDCA offer additive hepatoprotective effects.

The patient's high prognostic scores (including MELD) raised concerns about the need for liver transplantation.^25^ Ultimately, her favorable response to supportive care precluded this urgent intervention. Nevertheless, in cases of persistent hepatic enzyme elevation or unresolved coagulopathy refractory to maximal medical therapy, expedited transfer to a transplant center remains critical for improving survival odds.

This case highlights the clinical complexities arising from the intersection of severe dengue and AT‐DILI in endemic regions. Prompt recognition of hepatotoxicity signs during ATT, rapid cessation of implicated agents, and intensive supportive care remain crucial for optimized outcomes. While NAC's definitive benefit warrants further study, our case supports its consideration as a potential adjunct in similar presentations.

## AUTHOR CONTRIBUTIONS

**Naveen Gautam:** Conceptualization; project administration; resources; validation; writing – original draft; writing – review and editing. **Nishan Shrestha:** Conceptualization; writing – original draft; writing – review and editing. **Sanjeev Bhandari:** Supervision; writing – review and editing. **Sabin Thapaliya:** Supervision; writing – review and editing.

## FUNDING INFORMATION

No external funding was received.

## CONFLICT OF INTEREST STATEMENT

The authors declare that there are no conflicts of interest regarding the publication of this paper.

## CONSENT

Written informed consent was obtained from the patient's parent to publish this report in accordance with the journal's patient consent policy.

## Data Availability

The data are available from the corresponding author upon reasonable request.
